# Ecology and ethnoecology of dusky grouper [garoupa, *Epinephelus marginatus *(Lowe, 1834)] along the coast of Brazil

**DOI:** 10.1186/1746-4269-4-20

**Published:** 2008-09-15

**Authors:** Alpina Begossi, Renato AM Silvano

**Affiliations:** 1 Fisheries & Food Institute, Rua Coronel Quirino 1636, Campinas, S.P. 13025-002, Brazil; 2 Capesca, Preac-Cocen, Unicamp, C.P. 6194, Brazil; 3 Dept. Ecologia, UFRGS, Porto Alegre, Brazil

## Abstract

Dusky grouper (garoupa, *Epinephelus marginatus*) is an important catch for several artisanal small-scale fisheries along the Brazilian coast. It is a sedentary, monandric, and late maturing protogynous species, which makes it vulnerable to overharvesting even though it is mainly caught through hook and line or spear fishing through free diving. Lack of information on the ecology and biology of this species in Brazil is astonishing. Much of the information found in the literature concerns Mediterranean dusky groupers. Studies compiling local knowledge (ethnoecology) about fish species complement biological data, and have been fundamental for effective fisheries management. In this study, our objectives are to obtain data about dusky grouper through fish catches and analysis of stomach contents and gonad maturation (macroscopic analyses), along with interviews from fishermen from six small-scales communities from the southern (Pântano do Sul, Florianópolis, Santa Catarina State) to the northern Brazilian coast (Porto Sauípe, Bahia State). We conclude that precautionary approaches and 'data-less' management approaches are needed in the coast of Brazil. Research on this species and on the potential of aquaculture for its cultivation, are urgent, due to the apparent vulnerability and decrease of dusky grouper along the coast of Brazil.

## Introduction

The dusky grouper *(Epinephelus marginatus) *or garoupa, as it is called in Brazil, is widely distributed in the Atlantic, from the Mediterranean Sea to South Africa and Madagascar [1]. In the Western Atlantic this fish occurs in Brazil, with its southernmost distribution in Argentina [1]. In Brazil, it is caught frequently in artisanal fisheries of the southern coast, Santa Catarina State and the southeastern coast (São Paulo and Rio de Janeiro States) [2]. Being a highly prized fish, it is also sold and consumed locally [3-6]. Dusky grouper lives in rocky substrates and is one of the most important commercial fishes found on the southeastern Brazilian coast [7]. It occurs from shallow to deep waters, reaching a depth of more than 200 meters [2]. Much of the commercial catch is taken during summer spawning aggregations at specific sites known to fishermen [8].

Dusky grouper, is a monandric protogynous hermaphrodite. Female maturity occurs when females are 5 years old and 380 mm long (sub-mature females can weight 2–3 kg). Sex inversion, from female to male, occurs between the 9^th ^and 16^th ^years, peaking at 12 years, at a body weight of 9 kg [9]. Maturation of this fish occurs at body lengths of 350600 mm, according to data obtained in the area of Santa Catarina, southern Brazil [2].

Dusky grouper is considered to be overexploited in Mediterranean areas, in spite of the lack of statistical data on this species [8]. The lack of information about this important commercial fish species in Brazil is astonishing, especially considering its category of endangered status on the IUCN Red List. The Red List notes a decline of 88% in total catch, observed for seven countries [10]. Certainly, its slow growth and delayed maturity– contribute to the dusky grouper's susceptibility to commercial fisheries. Overall, reef fishes (groupers and snappers, for example) are heavily exploited and vulnerable to fishing pressure, possibly due their large body size, long life-span, slow growth, and late reproductive maturity [11]. In Brazil, there are some detailed surveys about the ecology and ethnoecology of another severely threatened species of serranid, the *Epinephelus itajara *or mero, but there are still few surveys addressing the biology or impact of fisheries of the dusky grouper along the Western Atlantic [2,12,13].

The local small-scale fisheries of reef fishes, such as groupers, are challenging to sustainably manage, largely because of their importance to both artisanal and recreational fishermen, the lack of resources available to implement management measures and the lack of biological data and resources for biological studies [14]. Studies recording and analyzing the local ecological knowledge of fishermen concerning exploited coastal fishes have been useful for better understanding local fishing practices, for complimenting conventional biological surveys and for gathering new biological information about fish migration, feeding habits and reproduction. These studies have also aided in marine conservation and revealed past abundance trends in the population of exploited fishes [15-20]. Therefore, fishermen's knowledge of fishing resources is especially important for supporting fisheries management in developing tropical countries, due to lack of research and biological data on exploited fishing resources on local and regional scales [21].

There is then, an urgent need to better understand the biology and ecology of the dusky grouper. The artisanal fishermen from the coast of Brazil have provided important ethnobiological information for several studies on other fish species [3,6,20,22]. For example, studies on the ethnobiology of *Epinephelus itajara *have contributed information on diet, reproduction, and equipment used in artisanal fisheries, among other aspects. Spawning aggregation of reef fishes is essential information for management, and can also be obtained through fishermen [12,13,15,23].

In this study, our objectives are to collect data on dusky grouper from the coast of Brazil, in particular fish landings data from two artisanal fisheries in SE Brazil: Bertioga and Rio de Janeiro. We also include ethnoecological data about this fish, including information on diet, habitat, and spawning seasons from these fisheries and other fisheries on the southeastern and northeastern Brazilian coasts. Such an approach could provide useful data, which is needed to properly manage the fisheries of reef fish in Brazil and other tropical and subtropical countries.

## Materials and methods

**Figure 1:**
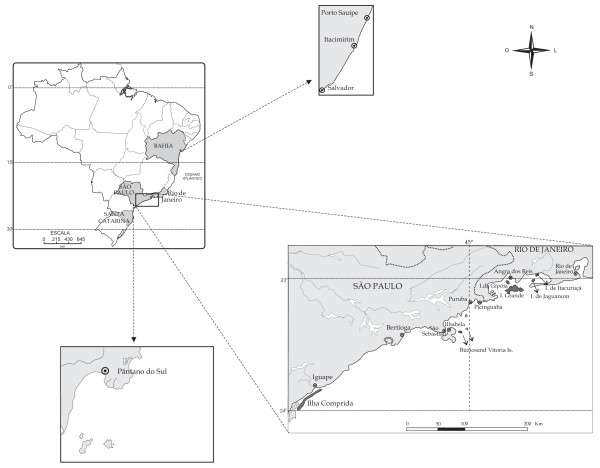
Map showing study areas in the coast of Brazil.

The methods employed in this study included collecting specimens of dusk grouper, marking fishing spots, and conducting two sets of interviews with local fishermen. Interviews were conducted in two different steps, from 2004 to 2006. The collection of specimens occurred in 2006–2007 from two landing points: Bertioga (São Paulo) and Copacabana beach ('Colônia de Pescadores do Posto 6', Rio de Janeiro, Figure 1). Dusky grouper was collected at landing points by buying the recently caught fish, which had been separated to be sold in the small local markets (Table 1). Such fish had their stomach contents analyzed and the presence/absence of mature gonads was observed. Such methods have been applied in other studies on marine fish species in Brazil [24]. Fish and crab identifications were performed by one of the authors (AB), and by L. S. Souza and L. E. C. Oliveira, and revised by R. Caires, and G. S. de Melo, respectively, from the Museum of Zoology, University of São Paulo; they were based on keys for Brazilian fishes and crabs [7,25].

**Table 1:** Number of individuals sampled of *Epinephelus marginatus *in the local market and landing point of Bertioga (coast of São Paulo) and Copacabana (coast of Rio de Janeiro), Brazil.

**Month (2006/2007)**	**Bertioga**	**Copacabana**
February	NS	5
March	NS	1
April	NS	5
May	NS	1
June	NS	0
July	NS	1
August	NS	2
September	NS	2
October	2	0
November	14	7
December	0	1
January	0	3
February	3	0
March	0	4
April	0	4
May	0	1
June	3	0
July	0	1
August	0	0
September	0	0
October	NS	NS
November	NS	2

**Total**	**22**	**40**

NS = no sampling in that locality.

**Figure 2:**
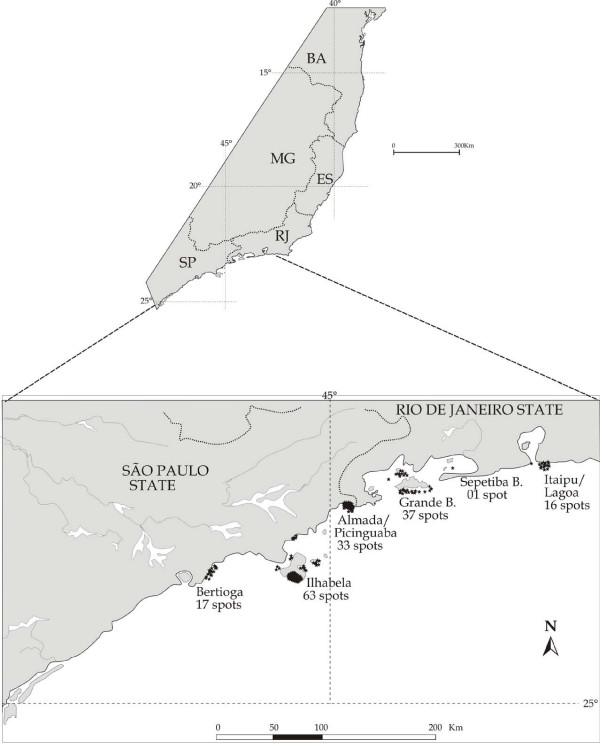
Some of the fishing spots used to catch groupers (garoupa) in the coast of Rio de Janeiro and São Paulo.

**Figure 3:**
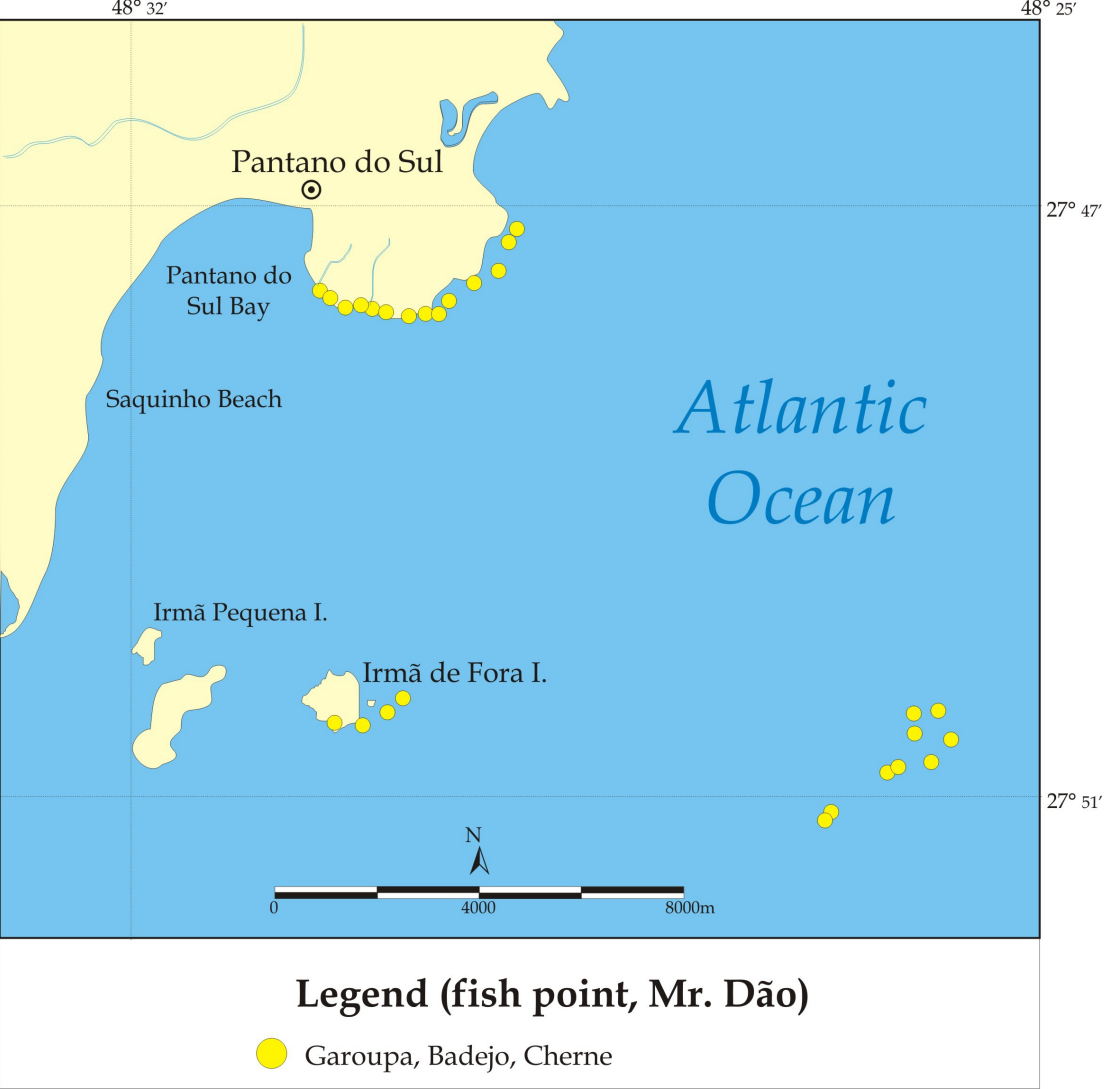
Fishing spots used to catch groupers (garoupa), including other groupers, such as other *Epinephelus *(cherne, mero) and *Mycteroperca *(badejo or comb grouper) at Pântano do Sul, Florianópolis, Santa Catarina State.

**Figure 4:**
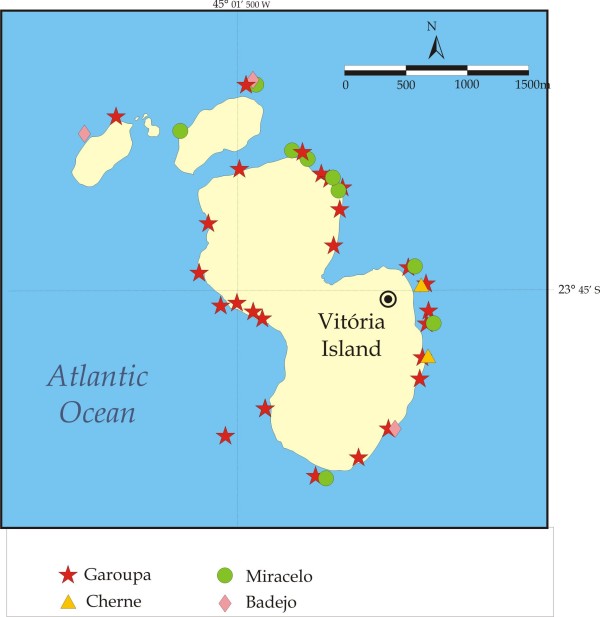
Fishing spots used to catch groupers, dusky grouper and other *Epinephelus *(cherne, mero), and *Mycteroperca *(badejo, miracelo or comb grouper) at Vitória Island, Parque Estadual de Ilhabela, São Paulo Coast.

**Figure 5:**
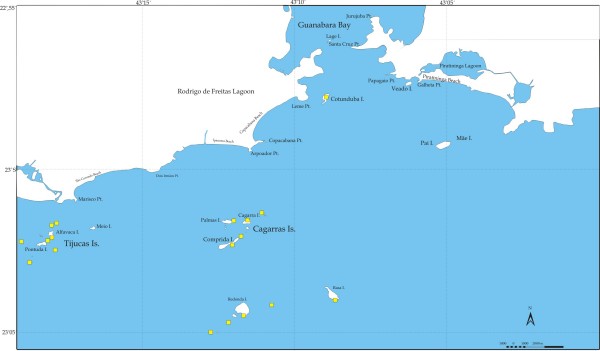
Fishing spots used to catch groupers in the city of Rio de Janeiro (Copacabana beach, *Colônia dos Pescadores do Posto 6*).

Fishing spots were mapped by boat, using GPS (Garmin III and V), with the help of experienced full-time fishermen (residents, with a minimum of 10 years fishing and living in the area) and locally appointed as 'skilled fishermen' (during the interview process). Maps also include data from sampling taken since 1998 (Figures 2, 3, 4, 5).

Fishermen interviewed live along the Atlantic Forest coast of Brazil, and in the southeastern coast they are named *caiçaras*, descending from Native Indians and Portuguese colonizers. Usually they represent low income populations, living through fishing, but some of them include small-scale agriculture and tourism as other sources of income [6,26].

The first set of interviews was done by showing pictures of *E. marginatus *along with 37 other fish species to artisanal fishermen, and asking about its identification, nomenclature, diet and habitat. Ninety-two fishermen from five different artisanal fisheries on the coast of Brazil were interviewed between the years of 2004–2005. The five artisanal fisheries were located in Copacabana and Itaipu beaches (Rio de Janeiro), Pântano do Sul (Florianópolis, Santa Catarina), and Itacimirim and Porto Sauípe (Bahia)(Figure 1). The average age of the fishermen interviewed were 53 years old, and most of them have four years of study or less. Porto Sauípe, in Bahia included 36% of illiterates or functionally illiterates.

The second interview set, conducted in 2005–2006, was concentrated on experienced local artisanal fishermen, 40 years and older, who had fished and lived in the area for 25 years or more. These interviews included questions on diet, habitat, spawning season, migration, and bait used for fishing. Forty-nine fishermen were interviewed, from Copacabana (Rio de Janeiro), Bertioga and Vitória island (São Paulo), and Itacimirim and Porto Sauípe (Bahia). Fishermen have usually four years of study or less, and their average age were 57 years old.

## Study areas

Artisanal fishing and diet, along with other ecological and ethnoecological information have been researched for a long time in the areas included in this study [27]. Moving from south to north along the coast of Brazil, we studied the fishing community of Pântano do Sul (Florianópolis), followed by several communities located along the coast of São Paulo and Rio de Janeiro States. The communities studied were the following: Bertioga, Buzios and Vitória Islands, Picinguaba, Puruba, Grande, Gipóia, Jaguanum and Itacuruçá Islands, Itaipu and Copacabana (within the city of Rio de Janeiro); Itacimirim and Porto Sauípe in Bahia State (Figure 1).

All the communities studied have a relatively small number of fishermen, ranging from an estimated 10 at Puruba to 50 in most sites. Methods employed for fishing are mostly hook and line and set gillnets, in motorized or paddled canoes. Small boats are used in Picinguaba and Itacimirim, allowing fishermen to reach more distant fishing spots. Spear fishing through free diving (snorkeling) has recently been increasing in popularity among young fishermen from Rio de Janeiro and Bahia States. Targets are mainly fish, but squid and crustacean (especially lobster in Bahia State) are also targeted. Other available information on these small scale fisheries has already been published [4,6,26,28-31].

## Results

### Fishing spots used to catch dusky grouper along the coast of Brazil: number of interviews and fish sampled

Sixty-two dusky groupers were collected in the local markets, 22 at Bertioga and 40 at Copacabana, mostly (50% of individuals sampled) in the month of November (Table 1). The locations where the dusky grouper (garoupa) are frequently caught by fishermen are shown in Figure 2. The figure refers to various coastal artisanal fishing communities in Rio de Janeiro and São Paulo, and includes the number of fishing spots used per locality. Detailed maps of places where dusky grouper were caught at Pântano do Sul, Florianópolis, Santa Catarina, Vitória Island and Copacabana, together with spots used for other Serranidae are shown in Figures 3, 4 and 5, respectively.

No grouper fishing spots were identified by fishermen in Bahia State. We did not collect *Epinephelus marginatus*, either at Bahia (Porto Sauípe/Itacimirim), (where we only collected another species, *Epinephelus adscensionis*), nor at Itaipu (Rio de Janeiro – in this case fishermen stressed that dusky grouper can only be caught only in distant flat rocks locally called 'lajes'). The northernmost distribution of *E. marginatus *in Brazil is found for Rio de Janeiro State [7]. Probably, information on dusky grouper distribution, at least for Bahia, may refer to other species of *Epinephelus*, since fishermen often refer to 'garoupa' as more than one species of that genus. This phenomenon was observed in another ethnoichthyological survey involving fishermen from several localities along the Brazilian coast [6].

Figure 5 shows a map of where the dusky groupers were sampled at Copacabana (n = 40). No other map was made for the fish collected at Bertioga, since those points are already in Figure 2. Moreover, most groupers sampled at Bertioga were caught by fishermen at Búzios Island, 24 miles off Ilhabela (Figure 2), often through line fishing. At Copacabana, most fish sampled were caught by young divers, also from the Colônia de Pescadores do Posto 6, except for a few caught involuntarily by set gillnets.

### Weight-Length (TL) relationships

**Figure 6:**
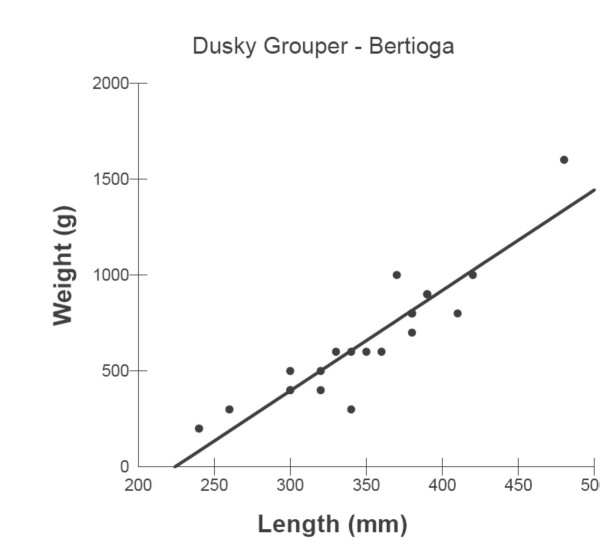
Weight-Total Length of *E. marginatus *collected at Bertioga.

**Figure 7:**
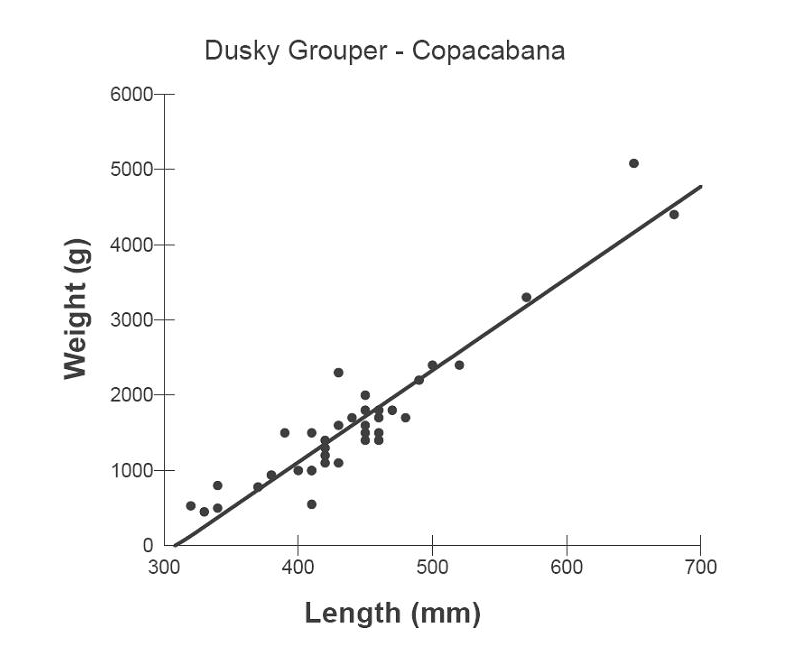
Weight-Total Length of *E. marginatus collected at *Copacabana.

Weight-Length (TL) relationships of the dusky groupers collected in Bertioga and Copacabana are shown through the relationship of Total Length (mm) to Weight (kg) in Figures 6 and 7, respectively. W-TL relationships are represented by the equations:

#### Bertioga

W = -1173.00 + 5.23TL, r^2 ^= 0.84, df = 21, p < 0.001 (Figure 6).

and

#### Copacabana

W = -3775.82 + 12.21TL, r^2 ^= 0.88, df = 36, p < 0.001 (Figure 7).

### Diet and reproduction of dusky grouper: analysis of stomach contents and of gonads

The stomach contents of the dusky groupers collected at Bertioga and Copacabana are shown in Table 2, which indicate the importance of crabs in their diet. No ontogenetic differences in the diet content were found between food items found in groupers at least at and above 400 mm. Crab prey were identified (Table 3); at Bertioga, most individuals found were *Petrolisthes galatinus *(Porcellanidae) and at Copacabana most were *Cronius rubber *(Portunidae). Gonads were not macroscopically visible, and we estimated they could be in the category F-1 (resting female) or J-1 (immature females), according to developmental stages for *E. marginatus *described in another study [8]. Nevertheless, we found mature gonads with visible eggs for two individuals of another species (*Mycteroperca acutirostris*, comb grouper or badejo), the gonads weighting 43 and 30 ml, at Copacabana, in January 2007.

**Table 2:** Stomach contents on sub-samples of *Epinephelus marginatus *collected in Bertioga (coast of São Paulo) and Copacabana (coast of Rio de Janeiro), Brazil.

Content	Bertioga	Copacabana	Total from the two localities	Total Length < = 400 mm	Total Length > 400 mm
Empty	9	12	21	11	10
Crabs	11	15	26	11	15
Fish^1^	4	12	16	7	8
Shrimp	0	3	3	0	3
Rest of food^a^	0	1	1	0	1
Total samples	22	39	61	27	32

^a ^Material mostly not identifiable, except for 2 sardines (Clupeidae) in the material from Bertioga.

Results are shown per class of total length: equal or less that 400 mm, and higher that 400 mm.

**Table 3:** Crabs identified in the stomach contents of *Epinephelus marginatus *from Bertioga (S.P.) and Copacabana (R.J.), Brazil^a^.

Locality	Crab Species	Family	No. individuals or rests found in stomachs
Bertioga	*Cronius ruber*	Portunidae	1
	*Eurypanopeus abbreviatus*	Xanthidae	1
	*Menipe nodifrons*	Majidae	1
	*Petrolisthes galatinus*	Porcellanidae	8
Copacabana	*Callinectes *sp.	Portunidae	1
	*Cronius ruber*	Portunidae	9
	*Eurypanopeus abbreviatus*	Xanthidae	2
	*Menipe nodifrons*	Majidae	1
	*Mithrax hispidus*	Majidae	2
	Not identified	Xanthidae	1

^a ^Material identified through the key by Melo (1996) and revised by Melo (MZUSP, June 2007)

### Local knowledge on dusky grouper (garoupa)

Dusky grouper is named locally by fishermen as 'garoupa'. In spite of having crabs as the principal stomach content at both Bertioga and Copacabana, fishermen's answers from both sets of interviews underestimated crabs in the diet of garoupa (Tables 4 and 5). Note, however, that when interviews were performed with skilled fishermen (Table 5), crabs are more often referred to (6%) compared to the less selective first set of interviews (4%, Table 4). Answers focused on fish are related to the bait fishermen use to catch garoupa, as observed in Table 6. Observe in Tables 4, 5, and 6 that fish, in particular bonito (Scombridae) and sardines (Clupeidae), are well represented both as bait and as answers regarding the diet of garoupa. The same results were found when questions were posed to buyers in the local fish market of Copacabana, at the time of the fish collection.

**Table 4:** Local Knowledge on the diet of *Epinephelus marginatus *in the coast of Brazil^a, b^

**Food Item**	**Copacabana**	**Itaipu**	**Pântano do Sul**	**Porto Sauípe**	**Itacimirim**	**Total**
Algae	1				1	**2**
Anchovy			7			**7**
Anything	2		3	1	1	**7**
Bonito	1	3	7			**11**
Crabs			2	2		**4**
Crustacean	4	1				**5**
Deteriorated fish/meat	2	1	1			**4**
Fish	5	2	8	22	7	44
Lobster				1		**1**
Meat	1					**1**
Mouse/snake			1			**1**
Octopus	2		2	1		**5**
Sardine	10	7	8	1	1	**27**
Shrimp	3		2	1	1	**7**
Small mollusks	2					**2**
Squid	4	5	9		1	**19**
Does not know				2	2	**4**
N	23	12	23	22	12	**92**

^a^Copacabana and Itaipu (Rio de Janeiro), Pântano do Sul (Florianópolis, Santa Catarina), and Itacimirim and Porto Sauípe (Bahia)

^b^Based on interviews showing 38 fish pictures, including *Epinephelus marginatus*.

**Table 5:** Local Knowledge on the diet of *Epinephelus marginatus *in the coast of Brazil^a, b^

**Food Item**	**Copacabana**	**Bertioga**	**Itacimirim**	**Pântano do Sul**	**Vitória sland**	**TOTAL**
Algae		1				**1**
Anything	3	3	3	7	1	**17**
Bonito	1	4	1	1	1	**8**
Crabs	1	2	1	2		**6**
Crustacean	2					**2**
Deteriorated fish/meat	1	5		7		**13**
Fish	2		15	8	2	**27**
Meat			1			**1**
Mouse/snake				1		**1**
Octopus		2			1	**3**
Sardine	3	6	2	2	1	**14**
Shrimp	1		2			**3**
Small mollusks	4	1				**5**
Squid		3		3		**6**
**Interviews**	10	11	15	11	2	**49**

^a^Bertioga and Vitória Island (São Paulo), Copacabana (Rio de Janeiro), Pântano do Sul (Florianópolis, Santa Catarina), and Itacimirim (Bahia).

^b ^Based don interviews with experienced fishermen [40 years up, fishing and living in the locality for 25 years or more]

**Table 6:** Baits used to catch *Epinephelus marginatus *in the coast of Brazil^a, b^

**Bait**	**Copacabana**	**Bertioga**	**Itacimirim**	**Florianópolis**	**Vitória Island**	**Total**
Anything	3	1		1		**5**
Artificial bait				6		**6**
Bird				1		**1**
Bonito	2	4		4	2	**12**
Crabs		3		1		**4**
Crustacea	2					**2**
Deteriorated meat	1	2				**3**
Fish		2	12	2		**16**
Maize grass				1		**1**
Meat				2		**2**
Octopus		2				**2**
Sardine	3	8	7	4		**22**
Scad			7			**7**
Shrimp	1	1	1			**3**
Small molluscs		1				**1**
Squid	1	2		4		**7**

^a^Bertioga and Vitória Island (São Paulo), Copacabana (Rio de Janeiro), Pântano do Sul (Florianópolis, Santa Catarina), and Itacimirim (Bahia).

^b^Based don interviews with experienced fishermen [40 years up, fishing and living in the locality for 25 years or more]

In both sets of interviews, fishermen considered the habitat of dusky grouper to be reefs or rocky substrates, particularly caves. Skilled fishermen stressed the great depths at which dusky grouper live (Tables 7 and 8). However, most of the citations quotations of dusky grouper in deeper places came from fishermen in northeastern Brazil (Table 8), and probably refer to other species of *Epinephelus*. It was noticed that in the northeastern Brazil fishermen go to deeper spots, since the continental shelf is narrow compared to the southeastern coast; its limits in Bahia are locally called ' paredão' (big wall).

**Table 7:** Local Knowledge on the habitat of *Epinephelus marginatus *in the coast of Brazil^a, b^.

**Habitat**	**Copacabana**	**Itaipu**	**Florianópolis**	**Porto Sauípe**	**Itacimirim**	**Total**
Cave	5	2		3		**10**
High depths	1		2	6		**9**
Islands	1		2			**3**
Open sea	1			6	1	**8**
Reefs/rocks	20	11	28	17	13	**89**
River				1		**1**
Sand/mud				3	1	**4**
Surface	1				1	**2**
Does not know				2	2	**4**
**Interviews**	23	12	23	22	12	**92**

^a^Copacabana and Itaipu (Rio de Janeiro), Pântano do Sul (Florianópolis, Santa Catarina), and Itacimirim and Porto Sauípe (Bahia).

^b^Based on interviews showing 38 fish pictures, including *Epinephelus marginatus*.

**Table 8:** Local Knowledge on the habitat of *Epinephelus marginatus *in the coast of Brazil^a, b^.

**Habitat**	**Copacabana**	**Bertioga**	**Itacimirim**	**Florianópolis**	**Vitória I.**	**Total**
Cave	2	1		3	2	**8**
High depths	2		18			**20**
Open sea			1			**1**
Reefs/rocks	7	11	6	12	2	**38**
Submersed ships				1		**1**
Surface			2			**2**
**Interviews**	**10**	**11**	**15**	**11**	**2**	**49**

^a^Bertioga and Vitória Island (São Paulo), Copacabana (Rio de Janeiro), Pântano do Sul (Florianópolis, Santa Catarina), and Itacimirim (Bahia).

^b^Based on interviews with experienced fishermen [40 years up, fishing and living in the locality for 25 years or more].

Only skilled fishermen were asked about spawning season, and about half did not know of the spawning season of dusky grouper (Table 9). Table 9 shows that 14 out of 24 fishermen believed that the spawning season of the dusky grouper occurs in the summer months in Brazil (November through March). Skilled fishermen were also asked about the migratory behavior of dusky grouper: 31 out of 49 considered it a non migratory fish, whereas 16 considered it a fish that migrates short distances to swim, eat or reproduce.

**Table 9:** Local Knowledge on spawning season of *Epinephelus marginatus *in the coast of Brazil^a, b^

**Spawning Season**	**Copacabana**	**Bertioga**	**Itacimirim**	**Florianópolis**	**Vitória I.**	**Total**
Apr–May	1					**1**
August			1			**1**
Cold/Rainy Season		3	3	1		**7**
December				2		**2**
Dec–Feb	1				1	**2**
Jul–May				1		**1**
June	1					**1**
March				1		**1**
Nov–Dec		1				**1**
November	1					**1**
Nov–May			1			**1**
Oct–Nov				4		**4**
Summer		2		1		**3**
Does not know	6	5	10	2	1	**24**
Interviews	10	11	15	11	2	**49**

^a^Bertioga and Vitória Island (São Paulo), Copacabana (Rio de Janeiro), Pântano do Sul (Florianópolis, Santa Catarina), and Itacimirim (Bahia).

^b^Based on interviews with experienced fishermen [40 years up, fishing and living in the locality for 25 years or more].

## Discussion

### Local fisheries: fishing spots, catch and local knowledge on reproduction

Places used to catch groupers in the study sites on the coast of Brazil coincide with the kinds of suitable dusky grouper habitat mentioned by fishermen: mostly island and rocky substrates. We do not have any information on the overlap in dusky grouper fishing areas between artisanal and recreational fisheries, since no data on recreational fisheries for dusky grouper are available. Nevertheless, such detailed data regarding the location of fishing spots are invaluable to future initiatives of coastal zoning and fisheries management. For example, marine reserves (no-take zones), where fisheries are forbidden, are one of the most effective tools for protecting reef fishes [32]. However, such reserves may represent considerable cost in terms of implementation, monitoring and social conflicts with fishermen. Sometimes marine reserves have been established without the necessary biological knowledge and without adequately considering the needs and concerns of affected local fishermen [33]. In such a context, our data on fishing grounds provides detailed information about regions important to fishermen and locations where possible conflicts could arise as a consequence of the establishment of protected areas.

Other studies of dusky grouper in other areas of the states of Bahia and São Paulo confirm the results regarding the habitat (rocky reefs) and the spawning season of dusky grouper that were obtained in the current study [6]. The greater availability of dusky groupers in Bertioga and Copacabana in November cannot be associated with spawning periods, since all were apparently immature females or resting females, since no mature gonads were found. However, such availability of dusky groupers to fishermen in late Spring (November) coincides with data obtained in Santa Catarina coast, where it was found a significantly higher density of dusky grouper juveniles in the Summer, together with a positive correlation of juvenile abundance and warmer water temperature [34]. Local knowledge on another species (mero – *Epinephelus itajara*) was collected in Babitonga bay, Santa Catarina, through interviews with local fishermen, recording that mero reproduces in January, but also that spawning aggregations occur in December [12]. This coincides with the information from fishermen in this study, suggesting that summertime is the also time of spawning for *E. marginatus*. Also in the Arvoredo Biological Marine Reserve, Santa Catarina, southern Brazil, *E. marginatus *matures in spring and spawns in early summer [2]. In the Mediterranean Sea, spawning of dusky grouper occurs in the summer [8]. Considering the typically high longevity and delayed maturity of fishes from the *Epinephelus *genus, the occurrence of only immature fish on fish landings observed in this study suggests possible occurrence of recruitment overfishing: individuals are caught before they are able to reproduce [11,35].

After a review of available ethnoecological studies on coastal and freshwater Brazilian fishes, results allowed hypotheses in order to compare local and scientific knowledge. 'Low likelihood' corresponded to unexpected hypotheses that contradicted existing biological data; 'medium likelihood' corresponded to hypotheses that could not be evaluated with the available scientific knowledge; and 'high likelihood' corresponded to hypotheses that agreed with available scientific data [36]. Hypotheses could then be derived about the dusky grouper (and other reef fishes) to be further tested based on local ecological knowledge of coastal fishermen from São Paulo and Bahia, respectively, at the southeastern and northeastern Brazilian coasts. Firstly, that the dusky grouper spawns in reefs, and secondly, that recreational divers may have been responsible for the population declines of this fish [36]. Comparisons about these hypotheses in the light of the available scientific literature, suggest that the first one would be unlikely and that the second would be of medium likelihood, as there are no available data for comparison [36]. Our results provide some support for the first hypothesis and data to test the second hypothesis, when comparable data on recreational fishing becomes available. However, not only recreational fishing, but also artisanal fishing, might be contributing to an apparent decrease in dusky grouper in the coastal waters of Brazil.

### Diet, growth, and local knowledge about dusky grouper and its fragility

The importance of knowing the diet of dusky grouper, *E. marginatus*, comes both from its high vulnerability to fishing pressure and from the limits of currently available studies on the diet of dusky grouper; these are based on a small number of stomachs or on a restricted range of fish sizes [37]. In our 61 samples of dusky grouper from Bertioga and Copacabana, crabs, followed by fish, were the most important food items found, and no ontogenetic differences were found concerning prey types. These results stand in contrast with ontogenetic differences found in another study showing that small (300 mm TL) dusky groupers feed primarily on crabs, followed by cephalopods (300–600 mm TL) and larger individuals feed on fish [37]. For reef fish species of other families, such as *Lutjanus griseus*, crabs increased in importance in the diet with increasing size of the fish [38].

The weight-length relationships of the dusky groupers collected at Bertioga and Copacabana (Rio de Janeiro) show that fish become relatively heavier for their length as they increase in size. Fish with a high value of K are heavy for their length, a result expected for a late maturing K species such as *E. marginatus *(K indicates a K-strategist, compared to an r-strategist) [39]. However, fish caught at both sites are usually between 300–450 mm (Total Length). This is the length of immature females, considering the protogynous characteristics of *E. marginatus*. Fish maturation size for this species in Brazil was estimated to be about 2 kg weight and 470 mm (Total Length) at which 50% of the females in the population reached sexual maturity (L_50_), with an amplitude of lengths of maturation between 350–600 mmm [2]. In the Mediterranean, the estimated mean length at first maturity (L_50_) is 438 mm (Standard Length) for females and 813 mm for males [8]. The minimum size for catches in Brazil, following the ' Instrução Normativa' 53 (November 22, 2005) of IBAMA (The Federal Agency for the Environment) from the MMA (Environment Ministry) is 470 mm. Taking into consideration the late maturity of *E. marginatus*, this catch size seems to be a 'non-precautionary approach' taken by the Government Environmental Agency. The relatively low size of the individuals caught in Bertioga and Copacabana suggests that juveniles are found in waters accessible to free divers (free diving using spear is one the most common methods of capture in Copacabana). Small sizes of *E. marginatus *(TL lower than 500 mm) have been found in shallow rocky areas, suggesting that newly settled individuals are recruited from nursery sites to shallow rocky areas [34]. Juveniles of dusky groupers have also been observed in rocky areas in the Mediterranean Sea [40]. Observed occurrences of small size groupers in the Mediterranean French coast, suggests that reproductive and spawning sites should be relatively close to the collecting areas [41]. Such observation is very relevant to consider the possibilities of protecting areas for groupers, following the example of European countries [42].

Dusky grouper has a slow growth length and late maturity, in addition to being monandric. Along with other reef fishes, the fragility of this species makes it highly vulnerable even to artisanal fisheries. Taking into account its high demand in local markets, where it is highly prized and an appreciated fish, its vulnerability is even higher. Caution should be taken towards harvest of slow-growing and late-maturing fish, even by low level artisanal fisheries [43]. In the Mediterranean region, especially in the French coast, *E. marginatus *is a protected species [41,42,44]. Studies on the genetics, reproduction, management, and aquaculture of *E. marginatus *are currently being undertaken by researchers in Europe. These studies offer advancements in the use of broodstocks for artificial spawning, amplifying the possibilities for the management and environmental manipulation of this species [45-48].

However, the aquaculture of carnivorous fish may have negative environmental and socioeconomic impacts. If the fish in culture are fed with other fish, the demand for these 'feeder' species may lead to declines in their population sizes, and thus render them less available to the local small-scale fishermen [14]. Our results indicating that juvenile (up to 400 mm) groupers feed mostly on crabs may provide another alternative for sustainable aquaculture, as reef-inhabiting crabs are not usually consumed or sold by fishermen from southeastern Brazil [4,30]. Furthermore, crabs may potentially be more easily reared in captivity in order to provide food for dusky groupers in aquaculture pounds.

## Conclusion

Due to the extreme vulnerability of dusky grouper (garoupa, *Epinephelus marginatus*) in Brazil and other areas, an intensification '*tour de force' *of studies using local knowledge and ecological knowledge should be undertaken, especially in the southeast and southern coast of Brazil, where most artisanal fisheries occur for this species. Research on the aquaculture of *E. marginatus *is recommended along with local management. Precautionary approaches and 'data-less' management approaches, such as the well-known examples from the Pacific, are also recommended for the Brazilian coast [16,17,21]. Following other authors, we stress that when long-term data sets are unavailable (as is the case for the Brazilian coast) skilled fishermen are an extremely valuable source of information [21].

Our results highlighted areas that are more intensively fished for dusky groupers by artisanal fishermen; in addition, they provide data on the diet and reproduction of this fish. Our approach is a cost-effective and feasible means of obtaining information about the fishery and biology of a threatened and commercially important reef fish species.

## Competing interests

The authors declare that they have no competing interests.

## Authors' contributions

AB conceived the study and carried out analyzes of stomach contents, identification of material, macroscopic analyses of the gonads, some interviews, and the marking of fishing spots. RAMS contributed within methods and performed interviews, besides contribution in the data analyses. Both authors read and approved the final manuscript.
